# StopWatch: The Preliminary Evaluation of a Smartwatch-Based System for Passive Detection of Cigarette Smoking

**DOI:** 10.1093/ntr/nty008

**Published:** 2018-01-24

**Authors:** Andrew L Skinner, Christopher J Stone, Hazel Doughty, Marcus R Munafò

**Affiliations:** 1 MRC Integrative Epidemiology Unit (IEU), University of Bristol, Bristol, UK; 2 School of Experimental Psychology, University of Bristol, Bristol, UK; 3 United Kingdom Centre for Tobacco and Alcohol Studies, University of Bristol, Bristol, UK; 4 Faculty of Engineering, University of Bristol, Bristol, UK

## Abstract

**Introduction:**

Recent developments in smoking cessation support systems and interventions have highlighted the requirement for unobtrusive, passive ways to measure smoking behavior. A number of systems have been developed for this that either use bespoke sensing technology, or expensive combinations of wearables and smartphones. Here, we present StopWatch, a system for passive detection of cigarette smoking that runs on a low-cost smartwatch and does not require additional sensing or a connected smartphone.

**Methods:**

Our system uses motion data from the accelerometer and gyroscope in an Android smartwatch to detect the signature hand movements of cigarette smoking. It uses machine learning techniques to transform raw motion data into motion features, and in turn into individual drags and instances of smoking. These processes run on the smartwatch, and do not require a smartphone.

**Results:**

We conducted preliminary validations of the system in daily smokers (*n* = 13) in laboratory and free-living conditions running on an Android LG G-Watch. In free-living conditions, over a 24-h period, the system achieved precision of 86% and recall of 71%.

**Conclusions:**

StopWatch is a system for passive measurement of cigarette smoking that runs entirely on a commercially available Android smartwatch. It requires no smartphone so the cost is low, and needs no bespoke sensing equipment so participant burden is also low. Performance is currently lower than other more expensive and complex systems, though adequate for some applications. Future developments will focus on enhancing performance, validation on a range of smartwatches, and detection of electronic cigarette use.

**Implications:**

We present a low-cost, smartwatch-based system for passive detection of cigarette smoking. It uses data from the motion sensors in the watch to identify the signature hand movements of cigarette smoking. The system will provide the detailed measures of individual smoking behavior needed for context-triggered just-in-time smoking cessation support systems, and to enable just-in-time adaptive interventions. More broadly, the system will enable researchers to obtain detailed measures of individual smoking behavior in free-living conditions that are free from the recall errors and reporting biases associated with self-report of smoking.

## Introduction

In a recent commentary, Naughton^1^ described the current knowledge on the potential for using mobile phones to deliver just-in-time (JIT) support for smokers attempting to quit. He detailed three types of JIT support: user-triggered support, in which the delivery of support is initiated by a request from the user, server-triggered support, which is initiated automatically on the basis of a set of pre-determined rules, and context-triggered support, which is delivered on the basis of detailed, dynamic information about the user, including their patterns of specific behaviors, location, and physiological state. Naughton described how using this rich context data can make support systems robust to within and between individual differences, and how the ultimate context-based system could be considered to be one that captures the necessary data for this unobtrusively, without the need for self-report.

One key item of data for a context-based smoking cessation support system will be a detailed measure of an individual’s smoking behavior. Naughton points out that the act of smoking can already be detected automatically using a wrist-worn accelerometer,^2^ and that this may soon be possible with off-the-shelf smartwatches. Here, we present a smartwatch system, called StopWatch, that does exactly this. It uses data from the motion sensors on a low-cost smartwatch to detect the signature hand movements of cigarette smoking, and does so without the need for a smartphone or data network connection.

A number of previous studies have explored different ways to use technology to passively detect cigarette smoking. One approach has been to use on-body sensors to measure respiratory rates, and to look for the patterns of changes in respiratory rate associated with cigarette smoking.^3^ This technique has been combined with the use of proximity detectors that measure hand-to-mouth movements to give increased sensitivity and specificity of smoking detection.^4^ Reliable measurement of respiratory rate, however, requires use of cumbersome thoracic sensor bands. These may be acceptable for use in short-term measurement sessions, but are not suitable for longer-term use in free-living conditions.

As motion sensors have become more commonplace in mobile and wearable digital devices, researchers began using these to detect the signature hand movements associated with cigarette smoking. Parate et al.^2^ demonstrated this using their RisQ system, which comprised a bespoke motion-sensor equipped wristband wirelessly connected to a smartphone. The wristband contained an integrated inertia measurement unit (IMU) that fused linear motion data from an accelerometer and angular motion data from a gyroscope with orientation data from a compass to provide three-dimensional trajectory data describing hand movements. These data were transferred to the smartphone by Bluetooth, and machine learning techniques were applied to classify instances of cigarette smoking.

The RisQ system achieves high level of sensitivity and specificity in laboratory and free-living tests, but it has the limitation that it requires a bespoke sensing wristband. Using this will be less arduous than a thoracic band respiratory sensor, but it is still an additional sensing device that places burden on the user. This issue has recently been addressed by a smoking behavior change system that uses the motion sensors in commercially available smartwatches and activity monitors to measure smoking behavior. SmokeBeat^5^ uses accelerometer and gyroscope data from a smartwatch or activity monitor, a smartphone application, and cloud-based analytics to detect the hand movements associated with cigarette smoking, identify patterns in their smoking behavior, and engage the user with behavior change techniques (eg, goal setting).

While SmokeBeat improves on RisQ in not requiring additional, bespoke sensing hardware, in its current form as an integrated system for smoking behavior change, it requires wireless connection to a smartphone to measure smoking behavior. While smartphone ownership continues to increase world-wide, ownership is still much lower among individuals with lower incomes (eg, in the United States, 64% for individuals earning <$30k, compared with 83% for those earning >$50k^6^). Low income is associated with higher prevalence of smoking globally,^7^ meaning that interventions aiming to reach sections of society with higher rates of smoking cannot make assumptions about smartphone ownership. Furthermore, those individuals with smartphones will typically only be within close proximity (and therefore wireless network range) of their smartphone approximately 90% of the time.^8^ This means that smoking detection systems relying on smartphones for detection will miss some aspects of smoking behavior (eg, leaving a smartphone inside when going outside to smoke).

StopWatch is a system that uses data from the motion sensors in a commercially available smartwatch, and identifies smoking events by applying machine learning methods that run entirely on the watch itself. Unlike RisQ it does not require the user to wear any bespoke sensing devices, and unlike RisQ and SmokeBeat, there is no need for a wirelessly connected smartphone. It is subject independent, and does not need to be trained to recognize an individual’s smoking gestures. It provides the potential for development of a number of smartwatch-based systems (that do not require smartphones), for helping smokers to quit, including a system enabling smokers to review their smoking behavior over time to better understand their patterns of smoking behavior, and JIT smoking interventions that use information about an individual’s patterns of smoking behavior to target more effective smoking behavior change interventions.

Here, we describe the implementation of the StopWatch system, and preliminary validation of the system in laboratory and free-living conditions.

## Methods

### System Overview

The StopWatch system comprises software that resides entirely within a low-cost, commercially available smartwatch. For development and validation, we used a G model watch from manufacturer LG, running the Android Wear v1.5 operation system. This device provided a good balance between battery life, comfort, and usability (essential for longitudinal use), an open development environment, and ease of access to sensor data (further details of smartwatch selection criteria are included in Supplementary Material). The accelerometer and gyroscope motion data were produced by an InvenSense MPU6516 IMU running in normal mode on the watch. These were sampled at a rate of 100Hz. The user interface for the StopWatch system is detailed in Supplementary Material.

### Analysis Pipeline

**Figure 1.**
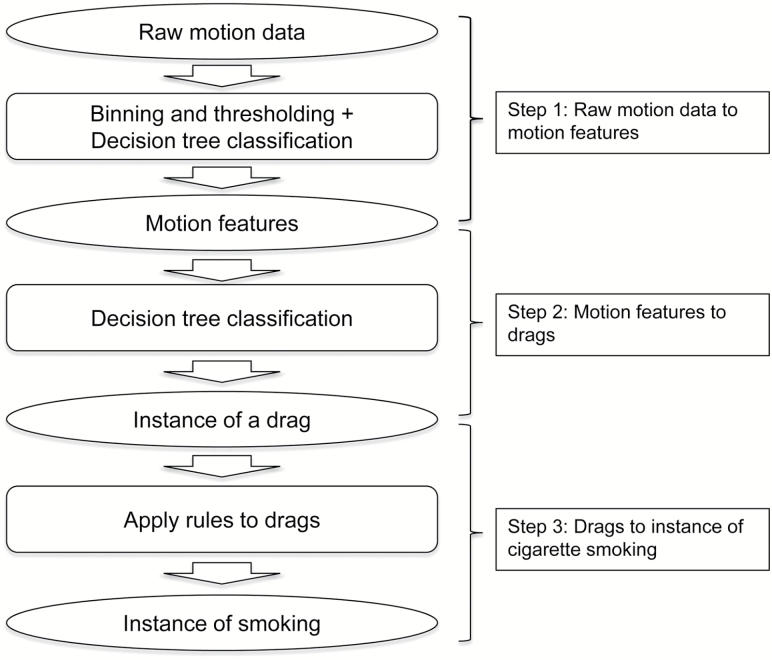
Analysis pipeline for detection of drags and instances of smoking.

Following the approach adopted by Parate et al.,^2^ a multi-stage analysis machine-learning pipeline was used to detect instances of cigarette smoking from raw motion sensor data. Unlike the Parate and SmokeBeat systems, our analysis pipeline runs entirely on a smartwatch, and not on a smartphone. We used a three-stage analysis pipeline, illustrated in Figure 1. *Step 1.* Raw motion data are subjected to binning and threshold (gyroscope data), and applied to an initial decision tree classifier (accelerometer data) to identify when a hand movement corresponds to one of a number of motion features relevant to smoking, which include “hand raising to mouth,” “hand stationary at mouth,” “hand moving away from mouth.” *Step 2.* Motion features are presented to a second decision tree classifier, which looks for features of particular values, happening in a specific pattern, to identify a single drag of a cigarette. Specifically, the decision tree looks for—hand raise to mouth motion that lasts between 0.3 and 0.7 seconds, followed by—hand stationary at mouth for between 0.4 and 8.0 seconds, followed by—movement of the hand away from the mouth. *Step 3.* The number of drags and time between drags is analyzed to look for a reliable instance of smoking a cigarette. When six drags are detected, with a duration of <80 seconds between drags, this is designated as an instance of smoking a cigarette.

The procedures for determining specific analysis pipeline parameters (based on laboratory and free-living smoking data collected from 38 participants), together with details of data formats and procedures for downloading smoking data from the StopWatch system are described in Supplementary Material.

### Validation and Results

Validation was performed using a set of 14 new smoking participants not previously involved in determining the parameters for the analysis pipeline (eligibility criteria are described in Supplementary Material). One participant was excluded from our validation data as it transpired that, contrary to the instructions provided to all participants, this participant had worn the smartwatch on their nondominant hand. The remaining 13 participants (6 female, all right-handed, mean age 21 years, SD 3 years) completed two stages of verification.

Firstly, system performance was assessed in a laboratory setting, with participants completing a number of tasks that included smoking a cigarette, drinking from a glass, and eating with hands and cutlery. All tasks were performed sitting down. Participants were first provided with detailed printed instructions, and a demonstration of the StopWatch system (they took the instructions with them after the laboratory session for reference in the free-living phase). The experimenter then moved to behind a two-way mirror to observe and record system performance during the different tasks. Overall, in this validation phase the system performed with a precision (the percentage of true positives among all the events identified by the system as smoking) of 75%, recall (the percentage of true positives among the actual smoking events) of 92%, and accuracy (the percentage of true positives and true negatives among the total number of events) of 90%. Further details of the laboratory validation results, and the methods used to compute the performance metrics, are included in Supplementary Material.

Participants subsequently took the system away and wore it in free-living conditions for a period of 24 hours. In this second phase, an adapted version of the application used previously to label motion data when identifying the analysis pipeline parameters, was used to record self-report data. With this, if the system failed to detect an instance of smoking, the participant could easily record this false negative with a button press on the smartwatch. Similarly, if the system detected an instance of smoking when the participant was not smoking, the participant could log this as a false positive with a single button press. (Note: this application was also running in the laboratory validation session to ensure no differences between the systems under test.) Participants also completed a paper diary of smoking events, recording the time and date of every cigarette smoked, and every false positive and false negative. In line with established techniques for testing classification system performance in extended free-living conditions, true negatives were not recorded, as these can artificially inflate performance statistics. This means accuracy cannot be determined, and system performance is instead characterized by recall and precision.

**Figure 2.**
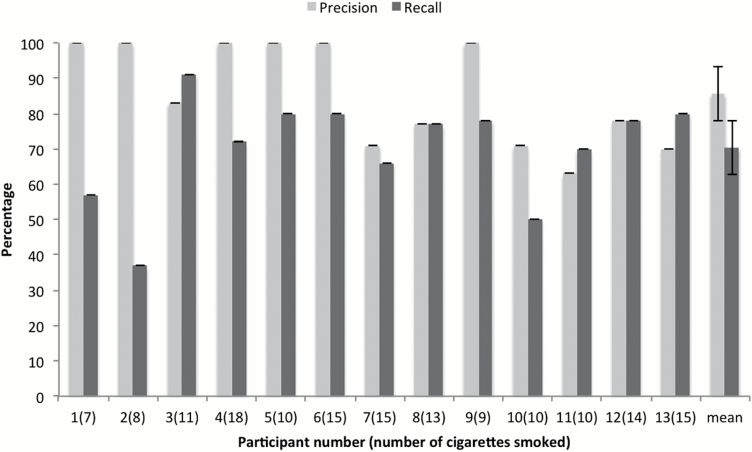
Free-living validation results.

A summary of the results of the free-living validation is shown in Figure 2. Overall, in the free-living validation phase the system performed with a precision of 86% (95% CI: 78% to 93%) and a recall of 71% (95% CI: 63% to 78%). As can be seen from Figure 2, there was considerable inter-participant variation in precision and recall. Performance data for the RisQ system (the only comparable system with detailed performance data available at this time) also shows notable variation in performance between participants. The variation in the StopWatch performance data is different to that observed in the RisQ data (more variation in recall performance with StopWatch and more variation in precision with RisQ), but this is to be expected as RisQ uses different machine learning methods. To explore the level of agreement between the data from StopWatch and the paper diaries, we calculated Cohen’s Kappa for each participant, which indicated substantial agreement between StopWatch data and diary data (mean 0.73, 95% CI: 0.67 to 0.79).

## Discussion

StopWatch is a system for passive detection of cigarette smoking. It uses data from the accelerometer and gyroscope motion sensors in a low-cost, Android smartwatch, and applies an analysis pipeline running on the watch to automatically detect and log instances of cigarette smoking. Preliminary validation of the system was performed using an LG G-Watch, running the Android Wear 1.5 operating system. In free-living conditions, the system achieved precision of 86% and recall of 71%.

We envisage a number of applications for the system. Because it detects smoking passively, requiring no input from the user, the system can provide detailed measurements of smoking behavior that are free from the recall errors and reporting biases associated with self-report of smoking.^9–12^ The system will therefore provide new opportunities for any researchers interested in measuring detailed patterns of smoking behaviors in individuals in free-living conditions, with minimal user burden, and at low cost.

Returning to the JIT smoking cessation support Naughton described, the StopWatch system provides the capability to unobtrusively capture smoking behavior data for context-triggered JIT support systems. Indeed, by gathering detailed smoking behavior data for individuals, StopWatch could enable more advanced forms of JIT support, such as Just-In-Time Adaptive Interventions.^13^

Other systems, like SmokeBeat and RisQ, also provide capability for passive detection of smoking. What sets StopWatch apart from these other systems is that it just uses a low-cost smartwatch, and does not require bespoke sensing hardware, a smartphone, or data network connectivity. This has several benefits: (1) The system will work as long as the smartwatch is worn and has power, and will not stop working if the watch is out of range from a paired smartphone or if it loses data network connectivity. (2) Using a commercially available smartwatch means we leverage the manufacturer’s investment in usability and design. This is important because for measurement and intervention systems that need to be worn and used for extended periods of time, user experience is an important consideration.^14^ (3) Using just a smartwatch keeps the cost of the system low. The watch we used is currently available for less than $100, and this (excluding the need to perform a brief set-up to load the application software onto the smartwatch), is the total cost of the StopWatch system.

Looking to the future, recent forecasts from wearable market experts indicate mobile network (cellular) connectivity will be one of the key new features that will see the market for smartwatches grow strongly in the next few years.^15^ Indeed, the wearable market is already seeing significant changes, with sales in basic activity monitors that cannot run third party applications declining, and sales of smartwatches showing substantial growth.^16^ The inclusion of mobile network connectivity is important, as it will increase the number of apps that can run on a smartwatch without the necessity to be paired with a smartphone. This is likely to shift the way smartwatches are used in the future, with users increasingly expecting a smartwatch app to be a standalone experience, free from the need for a smartphone.

In its current form, the StopWatch system has a number of weaknesses. The performance is not as high as other passive detection systems that use smartphone-based analysis pipelines (eg, Parate et al.^2^). For some applications, the current level of performance may not be an issue. Having modest recall means the system may miss some instances of smoking, but the high precision means when it does label an event as smoking, there is a good level of certainty the event was an instance of smoking. In the future, the processing power of smartwatches will increase, and it will be possible to run increasingly powerful classification algorithms on the watch, increasing both recall and precision performance. Another limitation of the system is that, while it will run on any Android smartwatch equipped with an accelerometer and gyroscope, it has currently only been validated running on an LG G-Watch.

Future work on the StopWatch system will include validating the system on a range of different smartwatches, exploring ways to increase the performance of the system, testing the feasibility of using the system in a variety of smoking behavior change interventions, and exploring the feasibility of using the system to passively measure use of electronic cigarettes and distinguish between cigarette smoking and electronic cigarette use in dual use individuals.

## Funding

This work was supported by the Medical Research Council Integrative Epidemiology Unit at the University of Bristol which is supported by the Medical Research Council and the University of Bristol (grants MC_UU_12013/6 and MC_UU_12013/7).

## Declarations of Interest

*The authors are listed as inventors on patent application PCT/GB2017/050110 “Method and Device for Detecting a Smoking Gesture”*

## Supplementary Material

Supplementary Material
