# Supplementary material for: StopWatch: The Preliminary Evaluation of a Smartwatch-Based System for Passive Detection of Cigarette Smoking
Source: Nicotine Tob Res. 2018 Jan 24;21(2):257–61. doi: 10.1093/ntr/nty008 (PMC6042639; doi:10.1093/ntr/nty008)
Supplement: Supplementary Material [file nty008_suppl_supplementary_material.docx]

Supplementary Material

**Smartwatch selection**

Information about smartwatch options was gathered from a variety of sources including manufacturer’s advertised specifications and consumer review websites (e.g., gadgetsandwearables.com). Battery life was assessed from manufacturer’s specification quoted in mAh, and usable hours from review sites; comfort was assessed by size, weight, form factor and review website reports; price was compared using prices quoted by a UK high-street retailer (John Lewis) and a leading retail website (Amazon.com) in November 2016.

**Determining analysis pipeline parameters**

Initially, we captured raw motion data and activity ground truth data (start and stop times) from 8 individuals as they smoked cigarettes and performed a number of other activities in both sitting and standing positions. Specifically, eating and drinking activities were included, as these have hand movements similar to smoking and so are likely to result in false positives. The raw motion data from the smartwatch were recorded on the smartwatch, and the ground truth data were recorded by an observer. The decision tree in step 1 was automatically generated from this motion and ground truth data, using the C4.5 algorithm ([an algorithm used to generate a decision tree](https://en.wikipedia.org/wiki/Decision_tree_learning) [from a set of training data)](https://en.wikipedia.org/wiki/Decision_tree_learning) with a pruning confidence of 0.25 in order to avoid over fitting to the training data. ^1^ The decision tree in step 2 was much simpler, and created manually from observation of the ground truth data.

Subsequently, parameters were refined through an iterative process of updating and testing on another 30 individuals as they smoked in free-living conditions. In this case, the smartwatch system was used to record the predicted start and stop time of smoking each cigarette, and individuals used a separate application running on the smartwatch to capture ground-truth data for these events.

**StopWatch User Interface**

The entry point for the StopWatch system user interface is the main screen, which is shown in Figure S1.

Figure S1. Stopwatch main screen

| Ready to detect  Number of drags: 0  Cigarettes today: 0  Battery level: 90% |
| --- |
| PRESS HERE TO RECORD AN UNDETECTED CIGARETTE |

When potential smoking movement is detected, the display changes, as shown in Figure S2, and the number of drags increments.

Figure S2. Display during potential cigarette detection

| Detecting  Number of drags: 1  Cigarettes today: 0  Battery level: 90% |
| --- |
| CURRENTLY ANALYSING  HAND MOVEMENTS |

If the movement is not classed as a smoking a cigarette, detection finishes, and the display returns to the main menu. If the system determines that a cigarette has been smoked, the watch vibrates, and a message is displayed, as shown in Figure S3.

Figure S3. Cigarette detected display

| Ready to detect  Number of drags: 6  Cigarettes today: 1  Battery level: 90% |
| --- |
| SMOKING DETECTED –  PRESS HERE IF YOU WEREN’T SMOKING |

If the participant has smoked a cigarette and the “smoking detected” message is correct, no further action is taken, and the message disappears after a couple of minutes and the user is returned to the main menu. If the participant has not smoked a cigarette and the smoking detection is therefore in error, the participant presses the red ‘Press if you weren’t smoking’ message box at the bottom of the screen. This removes the “false” cigarette from the database and displays the message shown in Figure S4. Swiping this screen away to the right returns the user to the main menu.

Figure S4. Cigarette disregarded display

| Smoking Detection Disregarded  Cigarettes today: 1  Swipe right to continue |
| --- |

If the participant has smoked a cigarette and the watch has not detected it, the participant presses the blue ‘Record undetected cigarette’ message box at the bottom of the main menu. The watch vibrates and the “cigarettes today” count on the main menu is incremented.

**Data format and exporting data**

Raw motion data are not stored on the watch; instead, details of puffs and cigarette smoking events identified by the analysis pipeline are recorded. This allows for efficient use of storage on the smartwatch. The StopWatch system contains a SQLite relational database comprising of three tables, one for cigarette data, one for drag data, and one for recording event data. Each table has an ID field as its primary key; the ID values of the cigarette table (cigarette ID) and the event table (event ID) are auto-incremented to unique values on row entry, and the ID value of the drag table (drag ID) is incremented under program control such that the combination of cigarette ID and drag ID is unique. Cigarette ID is referenced as a “foreign key” in the drag table to identify the relationship between a cigarette and its corresponding drags. The table structure is shown in Table S1.

Table S1. Data and SQLite database structure for StopWatch system.

| Table name: CIGARETTE | | | |
| --- | --- | --- | --- |
| Columns | CIGARETTE_ID | Primary key | Unique integer ID to identify cigarette |
|  | DATE |  | Date cigarette was smoked (YYYY-MM-DD) |
|  | CIG_TIME |  | Time cigarette was smoked (HH:MM:SS) |

| Table name: DRAG | | | |
| --- | --- | --- | --- |
| Columns | DRAG_ID | Primary key | Unique integer ID within table |
|  | CIGARETTE_ID | Foreign key | Primary key from CIGARETTE table |
|  | DRAG_TIME |  | Time drag was taken (HH:MM:SS) |
|  | START_TIME |  | System start time of drag (ns units) |
|  | END_TIME |  | System end time of drag (ns units) |

| Table name: EVENT | | | |
| --- | --- | --- | --- |
| Columns | EVENT_ID | Primary key | Unique integer ID to identify event |
|  | EVENT_DATE |  | Date of event (YYYY-MM-DD) |
|  | EVENT_TIME |  | Time of event (HH:MM:SS) |
|  | EVENT_DESC |  | Description of event (text) |

The event table allows for recording of system events under program control, for example gestures discounted because they were of too short or too long duration, battery level indications etc. For the validation trials, ground-truth data was recorded in this table.

Exporting of the smoking data is achieved by running a program to download data from the database into a local text file. Once downloaded, the data can be easily visualised, or loaded into a package such as Excel for further analysis.

**Eligibility criteria for validation study**

The eligibility criteria for the validation study were that participants:

- Should be daily smokers, smoking at least 10 cigarettes per day (not e-cigarettes)

- Should be able to smoke with their right hand, and not have any mobility issues affecting their right hand or right arm

- Must not have any food allergies or intolerances

- Must be aged 18 or over

**Determining performance metrics**

The paper self-report data was compared with the watch data (both the automated smoking detections and recordings by the user of false positives/false negatives) to confirm that the data captured by the watch was correct, matching the date and time of the paper self-report log entries with the date/time-stamp of the watch data. A detection of cigarette smoking by the watch, backed up by a self-report of having smoked a cigarette, was taken as a true positive. A false detection by the watch, with a corresponding self-report (on the app and on paper) of the watch incorrectly detecting smoking when no smoking had taken place, was classified as a false positive. A self-report of smoking which had no corresponding watch detection data (or where the participant reported that the watch failed to detect a cigarette) was taken to be a false negative. The number of true negatives in free-living conditions is unknown, as the participants only recorded smoking-related behaviour and there could be in theory an infinite number of non-smoking events.

Precision, recall and accuracy were then calculated as follows:

Precision = true positives / (true positives + false positives)

Recall = true positives / (true positives + false negatives)

Accuracy = (true positives + true negatives) / (true positives + true negatives + false positives + false negatives)

(Accuracy was calculated for laboratory-based validation only)

**Laboratory validation results**

Details of the laboratory tests of the StopWatch system are shown in Table S2.

Table S2. Laboratory-based validation results.

| Participant | Smoking | Drinking | Eating (fingers) | Eating (cutlery) |
| --- | --- | --- | --- | --- |
| 1 | TP | TN | TN | TN |
| 2 | TP | TN | TN | TN |
| 3 | TP | FP | TN | TN |
| 4 | TP | TN | TN | TN |
| 5 | TP | TN | TN | TN |
| 6 | TP | TN | TN | TN |
| 7 | TP | TN | FP | FP |
| 8 | FN | TN | TN | TN |
| 9 | TP | TN | TN | TN |
| 10 | TP | TN | TN | TN |
| 11 | TP | TN | TN | TN |
| 12 | TP | TN | TN | TN |
| 13 | TP | FP | TN | TN |

*TP = True Positive, TN = True Negative, FP = False Positive, FN = False Negative*

**Reference**

1. Quinlan JR. C4.5: Programs for Machine Learning. 1993. California:Morgan Kaufmann.
